# Impact of Chronic Disease Self-Management Program on the Self-Perceived Health of People in Areas of Social Vulnerability in Asturias, Spain

**DOI:** 10.3390/healthcare12080811

**Published:** 2024-04-09

**Authors:** Ester García-Ovejero, Marta Pisano-González, Isabel Salcedo-Diego, Pilar Serrano-Gallardo

**Affiliations:** 1 Nursing Department, Faculty of Medicine, Autonomous University of Madrid, 28029 Madrid, Spain; pilar.serrano@uam.es; 2 National Centre for Epidemiology, Instituto de Salud Carlos III, 28029 Madrid, Spain; 3 General Directorate of Social and Health Care and Coordination, Ministry of Health of the Principality of Asturias, 33005 Asturias, Spain; 4 Research Group “Person-Centered Care” of the Research Institute of Asturias (ISPA), 33005 Asturias, Spain; 5 Puerta de Hierro Majadahonda University Hospital, 28222 Majadahonda, Spain; 6 Puerta de Hierro-Segovia de Arana Health Research Institute (IDIPHISA), 28222 Majadahonda, Spain; 7 Interuniversity Institute “Advanced Research on Evaluation of Science and the University” (INAECU), 28029 Madrid, Spain

**Keywords:** chronic disease, self-management, self-rated health, health status disparities, vulnerable populations, health education, community health nursing, lay leaders, intervention studies

## Abstract

The Chronic Disease Self-Management Program (CDSMP) focuses on a health promotion perspective with a salutogenic approach, reinforcing the pillars of self-efficacy. The aim of this study was to assess the impact of the CDSMP on Self-perceived Health (SPH) in disadvantaged areas of Asturias, España. The study included vulnerable adults with experience of chronic diseases for over six months, along with their caregivers. The intervention consisted of a six-session workshop led by two trained peers. SPH was evaluated by administering the initial item of the SF-12 questionnaire at both baseline and six months post-intervention. To evaluate the variable “Change in SPH” [improvement; remained well; worsening/no improvement (reference category)], global and disaggregated by sex multivariate multinomial logistic regression models were applied. There were 332 participants (mean = 60.5 years; 33.6% were at risk of social vulnerability; 66.8% had low incomes). Among the participants, 22.9% reported an improvement in their SPH, without statistically significant sex-based differences, while 38.9% remained in good health. The global model showed age was linked to decreased “improvement” probability (RRRa = 0.96), and the “remaining well” likelihood drops with social risk (RRRa = 0.42). In men, the probability of “remaining well” decreased by having secondary/higher education (RRRa = 0.25) and increased by cohabitation (RRRa = 5.11). Women at social risk were less likely to report “remaining well” (RRRa = 0.36). In conclusion, six months after the intervention, 22.9% of the participants had improved SPH. Age consistently decreased the improvement in the different models.

## 1. Introduction

Social determinants of health are the circumstances in which people are born, grow, live, work, and age, having an impact on their health [1]. The Social Determinants of Health Framework proposed by the World Health Organization categorizes them into two major groups: structural determinants (socioeconomic and political context and axes of inequality such as gender, social class, ethnicity, and occupation) and intermediate determinants (living conditions, lifestyle, psychosocial, and biological factors) [2]. When these circumstances are unfavourable, the prevalence and progression of multiple chronic diseases increase [3].

In high-income countries, one-third of individuals aged 16 or older report living with long-term diseases, with prevalence rates even higher among those belonging to the lowest income quintile [4]. The global burden of non-communicable diseases is on the rise [5]. Among the top ten conditions with the greatest absolute increase in disability-adjusted life years, six are related to chronic conditions that primarily affect older adults: diabetes, chronic kidney disease, lung disease, cancer, hearing loss, stroke, and heart disease [5].

Managing chronic conditions involves multiple elements [6] integrated over the years into various models of care [7,8,9]. All of them emphasize the importance of fostering active and empowered patients, alongside establishing robust community connections and leveraging its resources. In 1978, a peer-led arthritis self-management program was initiated at Stanford University, which later expanded to other chronic diseases such as HIV, Diabetes Mellitus, or cancer, involving not only individuals with the condition but also their caregivers [10]. This program, named the “Chronic Disease Self-Management Program” (CDSMP), has been adopted in the United States [11] and in over twenty countries [12], showing favourable results in both health and cost savings [13,14,15,16]. The program approaches topics from a health promotion perspective with a salutogenic focus. It focuses not solely on the problems but also on their solutions for improvement and well-being, favouring health outcomes and health literacy in populations with low income [17]. The dynamics established in the program reinforce the self-efficacy pillars of Albert Bandura’s psychological theory [18].

Within the EFFICHRONIC context, a European project coordinated from the Principality of Asturias and implemented in Spain, the United Kingdom, France, Italy, and the Netherlands, the CDSMP intervention is offered to individuals with chronic diseases in socially vulnerable areas and their caregivers, to evaluate its benefits on health [12]. One of the expected benefits is the enhancement of Self-perceived Health (SPH), an indicator inversely associated with health problems, risk of hospitalization, and mortality [19,20,21,22,23], valid for people from diverse socioeconomic backgrounds, with or without chronic conditions, and of both gender [24,25]. SPH is also useful for equity measurements [20].

Improvement in SPH was expected after participation in the CDSMP. The objective of this study was to determine the impact of the CDSMP intervention on the SPH of individuals with chronic conditions and their caregivers, considering the axes of inequality in terms of age, education level, gender, income, and migratory status. Furthermore, the study analysed the impact of family situation, housing circumstances, interpersonal relationships, and social support as intermediate determinants influencing Self-perceived Health.

## 2. Materials and Methods

A quasi-experimental study was conducted from January 2018 to June 2020 in Asturias, Spain.

Adults experiencing chronic conditions persisting for over six months and their caregivers were invited to participate provided they resided in or frequented socio-sanitary centres located in socially vulnerable areas, had their basic housing needs covered, were fluent in the local language, and met one or more of the following criteria: being over 65 years old and living alone, residing in nursing homes, incarcerated individuals, individuals undergoing rehabilitation, migrants, ethnic minorities, or individuals with low incomes or educational attainment. Those with cognitive impairment, active addictions, or major mental illness hindering participation in group dynamics were excluded [26,27].

The estimated sample of Spanish participants was 500 persons [12]. After mapping priority vulnerable geographical areas identified through two deprivation indexes (rural and urban), several recruitment strategies were employed through the public healthcare system and networking with social structures (local initiatives, non-government organizations, and associations). Opportunistic recruitment and snowballing through the participants themselves were also used [26,28].

The development of activities was promoted by the Asturias Regional Health Authority. The programme followed the CDSMP guidelines: each intervention consisted of six weekly sessions of two and a half hours each, in small groups of twelve to sixteen individuals, led by two trained facilitators. At least one facilitator was a layperson (an individual with chronic illness or a caregiver), and one was a healthcare professional. The program was conducted in community settings such as social centres, municipal facilities, associations, nursing homes, or prison facilities. The approved CDSMP course materials were used [26,29].

The topics addressed during the intervention were related to treatment (moderate physical exercise, medication management, healthy eating, and evaluation of treatment options), symptom control (positive thinking, relaxation, sleep improvement, diaphragmatic breathing), and communication skills and decision-making (effective communication, improving interactions with healthcare professionals) [29].

The following variables were collected:

(a) Sociodemographic: Sex (man, woman), age (in years), migration status (country of birth: Spain, other), and highest educational level (no education; primary; first cycle of secondary; second cycle of secondary; post-secondary non-tertiary; short-cycle tertiary education; diploma or university degree; master’s degree or doctorate), subsequently recategorized into three categories: primary or less; secondary; higher education.
(b) Social Variables: Socio-familial risk based on the validated Gijón Questionnaire [30], consisting of six items with five response options. Scores range from 5 to 25 points (less than 10 indicates no social risk; 10 to 14 indicates socio-family risk; >15 indicates socio-family problems). For the analysis, each item was dichotomized: Low income (yes, no—with the cutoff point corresponding to the average salary in Asturias [31]; inadequate housing (yes, no); living alone (yes, no); absence of social relationships (yes, no); absence of social support (yes, no) (Table S1—Supplementary Materials) and the overall result was categorized into two groups (“no social risk” (5–10 points) and “social risk or socio-family problems” (≥10 points)) [30]. Institutionalization was also assessed (no institutionalization, nursing home, prison facilities, alcohol rehabilitation centre), as well as the residential area (rural peasant, rural intensive, peri-urban peasant, peri-urban intensive, urban), which was subsequently recategorized (urban or peri-urban; rural) [32].
(c) Self-perceived Health (SPH): Through the question ‘In general, would you say your health is: excellent, very good, good, fair, or poor?’ [33]. SPH is a good indicator for the study of health inequalities and especially in people with chronic conditions [19,20,21,22,23,24,25]. The ‘change in SPH’ was calculated from the subsequent measurement compared to the baseline, generating four categories [“improvement”; “worsening”; “no improvement” (for those starting at fair or poor categories and remaining there); “remained well” (for those who stayed in good, very good, or excellent categories)]. For the multivariate analysis, the “worsening” and “no improvement” categories were combined, leaving three categories: “improvement”, “remained well”, “worsening or no improvement”.

The questionnaires were self-administered, with the assistance of a researcher or an assistant if needed during the first session. At six months, the questionnaires were delivered as indicated in the first measure: by post or at the place of residence. The application time was approximately 30 min.

Descriptive statistics—univariate and bivariate—and multinomial logistic regression models, both overall and disaggregated by sex, were conducted for the variable “Change in SPH,” with “worsening or no improvement” as the base category. Some variables required recategorization to obtain sufficient observations for generating the models. Variables with statistical significance (*p* < 0.20) in the bivariate regression and others relevant to the phenomenon according to the literature were considered in the multivariate models. The interpretation of statistical significance uses the *p*-value <0.5/confidence intervals of Relative Risk Ratios in all cases. Stata 17 was the statistical program used.

The project was approved by the Research Ethics Committee of the Principality of Asturias on 31 January 2017 (No. 20/17), and written informed consent was obtained from all participants. The study protocol was registered through the ISRCTN Registry (Ref. ISRCTN70517103) on 20 June 2018. Data can be requested through the contact information provided there [27].

## 3. Results

A total of 559 participants agreed to participate in the study and completed the baseline questionnaire, with 343 completing the follow-up questionnaire. The pre/post sample with complete data for the SPH variable consisted of 332 participants.

The average age of the participants was 60.5 years (Standard Deviation = 15.1) and 67.4% were women (n = 223). Of the participants, 78.2% (n = 255) had chronic diseases, while the rest were caregivers; 31.1% (n = 88) were at social risk [men 35.9% (n = 33); women 28.8% (n = 55)], and 2.5% (n = 7; all men) had social and family problems according to the Gijón scale; 66.8% (n = 205) had low income, 10.3% (n = 34) were immigrants, and 49.9% (n = 161) had primary education or lower; 79% (n = 267) were not institutionalized, and 63.8% (n = 208) lived in rural areas (Table 1).

At baseline, SPH was poor or fair in 37.9% (n = 126) of participants, 35.2% in men (n = 38) and 39% in women (n = 87). Regarding the change in SPH, 22.9% (n = 76; 95% CI = 18.5–27.8) of patients improved, 38.9% (n = 129; 95% CI = 33.6–44.3) remained well, 19.6% (n = 65; 95% CI = 15.4–24.3) did not improve, and 18.7% (n = 65; 95% CI = 14.6–23.3) worsened (Table 2). Tables S2–S4 in the Supplementary Materials provide a bivariate descriptive analysis of the change based on participant characteristics.

In the global **multinomial bivariate** regression, men had a higher probability of **improvement** [crude relative risk ratio (RRRc) = 2.10; 95% CI= (1.15–3.82)] compared to the “worsening or not improving” group, and so did individuals in prison facilities [RRRc = 2.53; 95% CI= (1.15–5.54)] or alcohol rehabilitation centres [RRRc = 3.75; 95% CI (1.19–11.78)] and those with low income [RRRc = 2.6; 95% CI (1.28–5.28)]. The likelihood of improvement decreased with age [RRRc = 0.96; 95% CI (0.94–0.98)]. Furthermore, caregivers had a higher probability of **remaining well** compared to those in the “worsening or not improving” group [RRRc = 2.13; 95% CI (1.25–3.66)], while the likelihood was lower for those with chronic conditions [RRRc = 0.41; 95% CI (0.23–0.76)], social risk [RRRc = 0.49; 95% CI (0.27–0.9)] and with increasing age [RRRc = 0.98; 95% CI (0.96–0.99)]. No statistically significant associations were found with immigration status, education level, living alone or in inadequate housing, or the absence of social relationships (Table 3). Figures S1–S3 in the Supplementary Materials graphically represent the results of this and the following regression analysis.

In the **multivariate analysis**, age remained statistically significantly associated with **improvement** [adjusted relative risk ratio (RRRa) = 0.95 to 0.98; *p* < 0.05). Being a caregiver approached statistical significance (RRRa = 0.44; *p* < 0.1), but this was lost once the model was controlled by the presence of chronic conditions. Persons at social risk were less likely to **remain well** than to “worsen or not improve” [RRRa = 0.42; *p* < 0.05] (Models 4 and 5). Low income increased the likelihood of improvement [RRRa = 2.33 to 2.25; *p* < 0.05] when the analysis was based on items of social risk criteria included in the Gijón Questionnaire (Models 1B and 2B) (Table 4).

In the **disaggregated** bivariate regression, **men** with low income [RRRc = 4.21; 95% CI = (1.42–12.47)] and younger age [age: RRRc = 0.95; 95% CI = (0.92–0.98)] had a higher probability of **improvement** than the “worsening or not improving” group. Younger men [age: RRRc = 0.97; 95% CI = (0.94–0.99)] and those living with others [RRRc = 4.49; 95% CI = (1.5–13.45)] were more likely to **remain well** (Table 5). In the multivariate analysis, a lower probability of **improvement** was observed with age [RRRa = 0.95; *p* < 0.05] and a lower probability of **remaining well** was observed in those with secondary or higher education [RRRa = 0.25 to 0.29; *p* < 0.05]. Those who lived with others were more likely to remain well [RRRa = 5.11 to 5.2; *p* < 0.05] (Table 6).

No characteristics were associated with **improvement** in **women** in the bivariate analysis, but age did show a decrease in the probability of improvement in the multivariate analysis [RRRa = 0.95 to 0.97; *p* < 0.05]. Likewise, in the bivariate regression, caregivers had a higher probability of **remaining well** than “worsening or not improving” [RRRc = 2.56 95% CI (1.38–4.76)]; those at social risk [RRRc = 0.38; 95% CI (0.18–0.79)] or with chronic conditions [RRRc = 0.40; 95% CI (0.20–0.79)] were less likely to remain well than to worsen or not improve (Table 5). Having social risk, as opposed to not having it, remained statistically significant in the multivariate analysis [RRRa = 0.36 to 0.38; *p* < 0.05] (Table 6).

## 4. Discussion

### 4.1. Population Represented

This study distinguishes itself from other research exploring the effects of the CDSMP intervention on individuals with chronic diseases and their caregivers by specifically encompassing participants facing more pronounced social vulnerability. The indicators of social risk, such as housing problems, people with no social relationships, and individuals in need of care who do not receive it, were slightly higher than in the general population of Asturias [34]. Many self-management studies focusing on individuals with low incomes often recruit participants from centres they frequently visit or from vulnerable areas. However, few of these studies report measures at the individual or family level [17]. In this study, 66% of the participants had low incomes. 

### 4.2. Change in SPH: Magnitude of the Results, Interpretation, and Scope of the Indicator

The baseline prevalence of poor SPH (37.9% overall, 35.2% in men, and 39% in women) was high compared to the general population of Asturias (overall 30.4%; men 26.3%; women 34.2%) and similar to people with low incomes at the national level (overall 38.6%; men 33.5%; women 42.9%), according to data from the latest National Health Survey in Spain (35). In any case, when the SPH variable is operationalized with the values “excellent, very good, good, fair, or poor,” as in this study, rather than “very good, good, fair, poor, or very poor” [35], lower results in poor SPH [4,36] are obtained. This suggests that the participants in EFFICHRONIC may have underestimated poor SPH, and consequently it might have an even greater magnitude. The effect of categorization has also been described in the Latin population of the United States, where lower results are obtained if the category “fair” is translated to the Spanish questionnaire as “regular”—as in this case—rather than “passable” [37].

Among the various results evaluated in EFFICHRONIC, improvements have been reported in Self-perceived frailty [38], desires for lifestyle improvements, and a sense of community belonging [39], among others. In Spanish participants, six months after entering the program, 22.9% showed an improvement in SPH, and 38.9% remained well. In previous evaluations of CDSMP [40,41,42,43,44], the beneficial effect on SPH was observed in the short term, around four to six months, although there was no clear evidence of improvement one year after participation [40,41]. This effect occurs in other self-management improvement strategies [44,45,46]. In any case, the cited studies have continuously treated the SPH variable by calculating changes in the mean scores before and after the intervention, or exceptionally by calculating the change in the proportion of people with positive SPH results. This study represents the first assessment of changes in SPH by quantifying the improvement, maintenance, or deterioration for each participant subsequent to CDSMP intervention.

Utilizing non-specific indicators of specific diseases can prove valuable for facilitating comparisons of outcomes across various interventions [47]. In this regard, SPH is a good choice. In any case, selecting the outcome variable in studies of similar interventions with economically disadvantaged persons is not straightforward, and there are multiple options [48]. Schaffler found that effects on quality of life, self-efficacy, and empowerment were more often captured and yet, other physiological indicators, were captured less frequently [17]. On the contrary, Van Hecke found fewer reported significant effects from patient-reported outcomes and more from laboratory data [49]. The combination of both types of indicators is probably a useful strategy for future research. 

### 4.3. Changes in SPH and Axes of Inequality 

Getting older or ageing reduced the probability of improvement in different regression models, which has also been observed irrespectively of whether an intervention was received or not [19,50,51]. In the global bivariate analysis, a higher probability of improvement was found in men, which is expected based on the natural progression of SPH [51]. On the other hand, although a higher level of education has been associated with better health trajectories [51], in the present study it was found that SPH was less likely to be well maintained in men with a secondary or higher education level. It is possible that, by starting from a disadvantaged situation, men with a lower level of education could benefit more from an intervention that has been developed in a context of social vulnerability. In any case, this is an issue that needs to be studied in greater depth in future research. Other self-management intervention studies have found greater improvements in health-related quality of life in men, regardless of their educational level [52]; nevertheless, quality of life is a construct with more components than SPH. The disaggregated analysis, as required in previous studies [52], contributes to the understanding of the differential effect of self-management interventions in men and women.

### 4.4. Changes in SPH and Intermediate Social Determinants of Health

Material circumstances, along with psychosocial factors and behaviours, are factors that contribute to explaining socioeconomic inequalities in Self-perceived Health [53]. In the global analysis, individuals identified with social risk demonstrated a reduced likelihood of keeping well in terms of Self-perceived Health, a pattern also evident within the female subgroup models. In the case of men, those cohabiting with others were more likely to stay well, which is consistent with the literature reflecting that men living with companions report better Self-perceived Health [19,51]. Contrary to expectations, low income increased the likelihood of improvement in both the bivariate and multivariate analysis [51]. This result is valuable considering the goal of EFFICHRONIC, which aimed to improve the health of individuals in socially vulnerable situations. At the global bivariate level, a higher probability of improvement was also observed in people institutionalized in prisons and alcohol rehabilitation centres. While incarceration initially entails acute stress, requiring significant adjustments to relationships and sleep patterns, as well as chronic stress related to violent situations, harsh living conditions, or adaptation to internal social hierarchies [53], the study findings suggest that the CDSMP intervention may also act on these determinants and contribute to improving Self-perceived Health. Positive effects related to improving living conditions or treatments have been found in other studies [54].

Residential areas, which allowed for the examination of inequalities in the social determinants of health, did not show a statistically significant association with SPH. However, despite adhering to multiple criteria for geographical delineation, including population size and density, agricultural activities, soil characteristics, and, in certain cases, indicators of income [32], achieving precise categorization between rural and urban areas may have been challenging [55]. Differences in SPH between urban and rural areas do not consistently show a clear direction in various studies, and at least four categories and other variables such as deprivation level have been employed to delineate these disparities [56,57,58,59].

### 4.5. CDSMP Elements That Can Influence the Changes Found

CDSMP incorporates features frequently found in interventions for improving chronic disease self-management, which could favour results such as developing peer support through group meetings, providing educational resources and skills, developing a self-management plan, conducting interventions in community and healthcare settings, or involving community members who are trained alongside researchers and professionals [6,48,49,52]. The CDSMP intervention focuses on three tasks (disease management, role management, and emotional management) for which it trains five basic self-management skills: decision-making, problem-solving, effective use of available resources, improved healthcare practitioner–patient communication, and action planning [17,60]. The literature has already highlighted the beneficial role of problem-solving training in healthcare [17,49]. These skills can be useful for addressing common difficulties in the incorporation and maintenance of self-management of chronic conditions in daily routines [61], which could explain part of the improvement.

Although the program was implemented following its original design, personalization through simplified texts or images has not been shown to affect the effectiveness of self-management programs for vulnerable individuals (with low incomes or low literacy) [17,49]. Future studies could consider incorporating more individualized actions, such as appointment accompaniment, financial assistance, or extending the follow-up period [6,48,62], although this would require additional external resources.

A distinctive feature was the development and evaluation of a self-management program for individuals with chronic conditions within prisons, which has intrinsic value as it provides access to a rarely addressed scenario where programs are typically directed at communicable diseases or substance abuse, not chronic conditions [63].

On the contrary, in EFFICHRONIC, the CDSMP intervention activities were conducted in-person, which might have contributed to more favourable outcomes. The effectiveness of information technology-based strategies remains uncertain, both in CDSMP interventions [41] and in other programs [17,64]. However, concerning diabetes control among low-income and ethnic minority individuals, interventions involving physical meetings have yielded superior results [62].

Despite the inclusion of multiple favourable components in CDSMP, there are factors beyond the scope of such programs that may affect the outcomes, such as the severity of the disease or living conditions [65].

### 4.6. Limitations

While the number of individuals with complete SPH data available for analysis was lower than projected, the intended sample size for the study was reached, which is a challenging achievement in the context of vulnerable populations [66] and chronic disease programs [45]. A previous review identified several reasons that make recruitment challenging for CDSMP programs: rurality time constraints, which were overcome in this study by extending the study duration to achieve the necessary sample size, and cultural barriers, which may be related to the low response rate [67]. In addition, the evaluation of the final workshops (90 subjects) coincided with the COVID-19 pandemic, which might have contributed on the one hand to a loss of subjects for analysis and on the other hand to an underestimation of the beneficial effects of the intervention on the basis that COVID-19 had a large negative impact on health and on people’s lives in general [68]. Furthermore, both factors could have introduced a selection bias.

Additionally, there might have been an information bias due to the length of the questionnaire, and beyond the Hawthorne effect [69], as the personal relationship established with the research team during the intervention could have positively influenced the outcomes.

Although individual factors have been adjusted and controlled when evaluating the results, it must be considered that the included individuals belong to a single region, which should be considered when extrapolating the results to other contexts.

### 4.7. Recommendations and Clinical Implications

After CDSMP, almost a quarter of participants improved their self-perceived health and almost 40% remained in good health. However, social risk, especially in men living alone, decreases the effectiveness of the CDSMP. Therefore, this needs to be considered when planning interventions for chronic populations in contexts of social vulnerability.

### 4.8. The New Directions for Future Research

For future research on the CDSMP, it is advisable to consider including a control group, blinded data collection methods, shorter questionnaires to facilitate participation, and the inclusion of physiological measurements besides the patient-reported outcomes. There is also a need to evaluate the intervention with vulnerable people in other settings and to further assess the influence of educational level on the health outcomes.

## 5. Conclusions

After six months of the CDSMP activity in areas of social vulnerability, an improvement in SPH was observed in nearly a quarter of the participants, and nearly 40% maintained good health. Age consistently had the most significant effect in reducing the likelihood of improvement compared to “worsening or not improving”. Maintaining good SPH was less likely than worsening or not improving for individuals at social risk and as age increased. For men, they were more likely to maintain good health when living with someone and less likely if they had secondary or higher education. In women, the probability of maintaining good health, compared to worsening, decreased when facing social risk.

## Figures and Tables

**Table 1:** Description of the sample studied (global and disaggregated by sex).

		Global (n = 332; 100%)		Men (n = 108; 32.6%)		Women (n = 223; 67.4%)	
Variables		Mean (SD)	Min–Max	Mean (SD)	Min–Max	Mean (SD)	Min–Max
Age (n = 330)		60.5 (15.1)	18.3–89.9	55.7 (16.7)	19.9–87.7	62.8 (13.7)	18.3–89.9
Social risk (Continuous variable *) (n = 283)		8.69 (2.6)	5–18	9.2 (3.2)	5–18	8.4 (2.2)	5–14
**Variable**	**Categories**	**n**	**%**	**n**	**%**	**n**	**%**
Participant type (n = 326)	Chronic	227	69.6	87	82.1	139	63.5
	Caregiver	71	21.8	16	15.1	55	25.1
	Both	28	8.6	3	2.8	25	11.42
Being a caregiver (n = 326)	No	227	69.6	87	82.1	139	63.5
	Yes	99	30.4	19	17.9	80	36.5
Chronicity (n = 326)	No chronic disease	71	21.8	16	15.1	55	25.1
	With chronic disease	255	78.2	90	84.9	164	74.9
Migration status (Country of birth) (n = 329)	Yes (Other country)	3.4	10.3	13	12.3	21	9.5
	No (Spain)	295	89.7	93	87.7	201	90.5
Educational level (n = 323)	Primary or less	161	49.9	45	42.9	115	53
	Secondary	121	37.5	47	44.8	74	34.1
	Higher education	41	12.7	13	12.4	28	12.9
Institucionalization (n = 338)	None	267	79.0	51	47.2	209	93.7
	Nursing home	7	2.1	4	3.7	3	1.4
	Prison facilities	47	13.9	42	38.9	5	2.2
	Alcohol rehabilitation centre	17	5.0	11	10.2	6	2.7
Residential area (n = 331)	Rural Peasant	76	23.9	21	19.6	54	24.9
	Rural Intensive	132	39.9	57	72.9	74	59.0
	Peri-urban Peasant	43	13.0	8	7.5	32	73.7
	Intensive Peri-urban	43	13.0	8	7.5	3.4	89.4
	Urban	37	11.2	13	12.2	23	100
Social risk global assessment (Gijón Questionnaire *) (n = 283)	No social risk	188	66.4	52	56.5	136	71.2
	With socio-familial risk	88	31.1	33	35.9	55	28.8
	Socio-familial problems	7	2.5	7	7.6	0	0
Dichotomized Gijón questionnaire variables (social risk) **							
Low level of income (n = 307)	Yes	205	66.8	63	61.8	141	69.1
	No (More than 1060 euros per month)	102	33.2	39	38.2	63	30.9
Live alone (n = 324)	Yes	84	25.9	32	30.5	52	23.7
	No (They live accompanied with or without dependence)	240	74.1	73	69.5	167	76.3
Absence of social relationships (n = 326)	Yes (I do not leave the house or receive visitors (or less than once a week)	6	1.8	6	5.8	0	0
	No	320	98.2	98	94.2	221	100
Absence of social support (n = 312)	Yes (I need permanent care that is not provided)	2	0.6	2	2.0	0	0
	No	310	99.4	97	98.0	212	100
Inadequate housing (n = 327)	Yes	61	18.7	19	17.9	41	18.6
	No (It is appropriate to my needs)	266	81.4	87	82.1	179	81.4

* Gijón Scale: minimum score = 5 and maximum = 25 points. Sections: <10 indicates no socio-familial risk, 10 to 14 in risk, >15 problems ** The recategorized category is described alone in each variable, and the rest of the categories are included in the other option.

**Table 2:** Results of Self-perceived Health (global and disaggregated by sex).

		Global (n = 332; 100%)		Men (n = 108; 32.6%)		Women (n = 223; 67.4%)	
Variable	Categories	n	%	n	%	n	%
Self-perceived health baseline measurement	Poor	17	5.1	6	5.6	11	4.93
	Regular	109	32.8	32	29.6	76	34.1
	Good	147	44.3	48	44.4	99	44.4
	Very good	47	14.2	16	14.8	31	13.9
	Excellent	12	3.6	6	5.6	6	2.7
Self-perceived health post measurement	Poor	18	5.4	7	6.5	11	4.9
	Regular	95	28.6	23	21.4	71	31.8
	Good	158	47.6	50	46.3	108	48.4
	Very good	46	13.9	21	19.4	25	11.2
	Excellent	15	4.5	7	6.5	8	3.6
Change in self-perceived health	Worsening	62	18.7	19	17.6	43	19.3
	No improvement	65	19.6	16	14.8	48	21.5
	Remained well	129	38.9	39	36.1	90	40.4
	Improvement	76	22.9	3.4	31.5	42	18.8

**Table 3:** Bivariate multinomial regression of change in Self-perceived Health for the reference category “worsening or no improvement”.

		n	Remained Well (n = 129; 38.9%)		Improvement (n = 76; 22.9%)		
Variable	Categories		RRRc	95% CI	RRRc	95% CI	*p* Value
Age (n = 330)		330	0.98	0.96/0.99	0.96	0.94/0.98	0.001
Being a caregiver (n = 326)	No	227	1		1		0.001
	Yes	99	2.13	1.25/3.66	0.692	0.34/1.41	
Chronicity (n = 326)	No chronic disease	71	1		1		0.002
	With chronic disease	255	0.41	0.23/0.76	1.24	0.55/2.82	
Sex (n = 331)	Men	108	1.13	0.65/1.94	2.10	1.15/3.82	0.038
	Women	223	1		1		
Migration status (Country of birth) (n = 329)	Yes (Other country)	3.4	1		1		0.882
	No (Spain)	295	1.21	0.54/2.75	1.03	0.41/2.59	
Educational level (n = 323)	Primary or less	161	1		1		0.667
	Secondary or higher education	162	1.17	0.72/1.93	1.28	0.72/2.30	
Social risk global assessment (Gijón Questionnaire) (n = 283)	Risk free	188	1		1		0.005
	At risk or with socio-familial problems	95	0.49	0.27/0.9	1.36	0.73/1.37	
Institution (n = 325)	Elderly residence	7	Not included in this analysis *				0.005
	Prison facilities	47	1.06	0.49/2.28	2.53	1.15/5.54	
	Alcohol withdrawal centre	17	0.56	0.13/2.39	3.75	1.19/11.78	
	No	267	1		1		
Residence area (n = 325)	Rural	208	1		1		0.782
	Urban or peri-urban	123	0.83	0.50/1.40	0.88	0.49/1.60	
Low level of income (n = 307)	Yes	205	1.02	0.60/1.72	2.6	1.28/5.28	0.012
	No	102	1		1		
Live alone (n = 324)	Yes	84	1		1		0.167
	No	240	1.69	0.95/3.00	1.06	0.56/2.01	
Absence of social relationships (n = 326)	Yes	60	1		1		0.838
	No	320	1.55	0.25/9.43	1.77	0.18/17.34	
Inadequate housing (n = 327)	Yes	61	1		1		0.328
	No	266	1.54	0.80/2.95	0.96	0.48/1.94	

RRRc: Crude relative risk ratio. * Those institutionalized in a nursing home were eliminated due to the low number of observations.

**Table 4:** Multivariate multinomial regression models of change in Self-perceived Health for the reference category “Worsening or no improvement”.

**Models Using Global Assessment of the Social Risk Variable (Gijón Questionnaire)**												
	**Model 1**		**Model 2**		**Model 3**		**Model 4**		**Model 5**		**Model 6**	
	**Remained Well**	**Improvement**	**Remained Well**	**Improvement**	**Remained Well**	**Improvement**	**Remained Well**	**Improvement**	**Remained Well**	**Improvement**	**Remained Well**	**Improvement**
**Variable**	**RRRa** **(CI95% Min/Max)**	**RRRa** **(CI95% Min/Max)**	**RRRa** **(CI95% Min/Max)**	**RRRa** **(CI95% Min/Max)**	**RRRa** **(CI95% Min/Max)**	**RRRa** **(CI95% Min/Max)**	**RRRa** **(CI95% Min/Max)**	**RRRa** **(CI95% Min/Max)**	**RRRa** **(CI95% Min/Max)**	**RRRa** **(CI95% Min/Max)**	**RRRa** **(CI95% Min/Max)**	**RRRa** **(CI95% Min/Max)**
Age	0.98 * (0.96/0.99)	0.96 * (0.94/0.98)	0.98 * (0.96/0.99)	0.97 * (0.94/0.99)	0.97 * (0.95/0.99)	0.96 * (0.94/0.98)	0.98 ** (0.96/1.00)	0.95 * (0.93/0.98)	0.98 (0.96/1.00)	0.95 * (0.93/0.98)	0.88 (0.96/1.00)	0.96 * (0.93/0.98)
Sex (men)			0.98 (0.57/1.72)	1.68 (0.90/3.13)	0.89 (0.49/1.65)	1.51 (0.76/2.99)	1.11 (0.59/2.11)	1.36 (0.66/2.82)	1.1 (0.58/2.07)	1.34 (0.65/2.79)	1.07 (0.57/2.01)	1.54 (0.76/3.12)
Social risk global assessment (Gijón Questionnaire) (at risk)					0.41 * (0.22/0.76)	0.95 (0.48/1.87)	0.42 * (0.22/0.77)	0.8 (0.39/1.60)	0.42 * (0.23/0.79)	0.80 (0.39/1.62)	0.42 * (0.22/0.79)	0.82 (0.41/1.65)
Is a caregiver (yes)							1.73 (0.92/3.26)	0.44 ** (0.19/1.03)	1.17 (0.46/2.98)	0.33 (0.08/1.32)		
Chronicity (yes)									0.56 (0.20/1.56)	0.64 (0.14/3.02)	0.49 ** (0.25/1.00)	1.64 (0.64/4.21)
**Models Using the Individual Items of the Socio-Familial Risk Variable (Gijón Questionnaire)**												
	**Model 1B**		**Model 2B**		**Model 3B**		**Model 4B**		**Model 5B**		**Model 6B**	
	**Remained Well**	**Improvement**	**Remained Well**	**Improvement**	**Remained Well**	**Improvement**	**Remained Well**	**Improvement**	**Remained Well**	**Improvement**	**Remained Well**	**Improvement**
	**RRRa** **(CI95% min/max)**	**RRRa** **(CI95% min/max)**	**RRRa** **(CI95% min/max)**	**RRRa** **(CI95% min/max)**	**RRRa** **(CI95% min/max)**	**RRRa** **(CI95% min/max)**	**RRRa** **(CI95% min/max)**	**RRRa** **(CI95% min/max)**	**RRRa** **(CI95% min/max)**	**RRRa** **(CI95% min/max)**	**RRRa** **(CI95% min/max)**	**RRRa** **(CI95% min/max)**
Age	0.97 * (0.96/0.99)	0.97 * (0.95/0.99)	0.97 * (0.95/0.99)	0.97 * (0.95/0.99)	0.99 (0.97/1.01)	0.96 * (0.94/0.99)	0.98 (0.96/1.01)	0.96 * (0.94/0.99)	0.98 (0.97/1.01)	0.96 * (0.95/0.99)	0.98 (0.96/1.00)	0.97 * (0.95/0.99)
Sex (men)	0.99 (0.55/1.78)	2.12 * (1.08/4.14)	1.01 (0.56/1.82)	1.99 * (1.01/3.92)	1.25 (0.68/2.31)	1.79 (0.88/3.67)	1.23 (0.66/2.29)	1.79 (0.87/3.67)	1.20 (0.66/2.21)	1.94 ** (0.97/3.93)	1.19 (0.65/2.19)	2.06 * (1.03/4.13)
Low income (yes)	0.89 (0.52/1.55)	2.33 * (1.10/4.92)	0.87 (0.51/1.52)	2.25 * (1.06/4.75)	0.88 (0.50/1.54)	1.91 ** (0.90/4.09)	0.88 (0.50/1.55)	1.91 ** (0.90/4.09)	0.88 (0.50/1.29)	1.96 ** (0.92/4.20)	0.90 (0.51/1.57)	2.03 ** (0.95/4.34)
Live alone (Yes)			0.67 (0.37/1.23)	1.07 (0.53/2.17)	0.72 (0.39/1.33)	1.03 (0.51/2.09)	0.71 (0.38/1.30)	1.02 (0.50/2.07)	0.70 (0.38/1.29)	1.05 (0.52/2.14)		
Is a caregiver (yes)					1.83 ** (0.99/3.39)	0.56 (0.24/1.28)	1.16 (0.47/2.92)	0.53 (0.15/1.88)	--	--		
Chronicity (yes)							0.52 (0.19/1.40)	0.91 (0.22/3.79)	0.46 * (0.23/0.90)	1.54 (0.61/3.91)	0.45 * (0.23/0.88)	1.56 (0.612/3.97)

RRRa: Adjusted relative risk ratio. Reference categories: women; without social risk; not low income; not living alone; not a caregiver; does not have chronic disease. * *p* value < 0.05 ** *p* value 0.05–0.1.

**Table 5:** Bivariate multinomial regression of change in Self-perceived Health for the reference category “Worsening or no improvement” in men and women.

		Men						Women					
			Remained Well		Improvement				Remained Well		Improvement		
Variable	Categories	n	RRRc	95% CI	RRRc	95% CI	*p* Value	n	RRRc	95% CI	RRRc	95% CI	*p* Value
Age		107	0.97	0.94/0.99	0.95	0.92/0.98	0.000	222	0.98	0.96/1.01	0.97	0.95/1.00	0.174
Social risk (Continuous *) (n = 92)		92	0.91	0.77/1.07	1.04	0.89/1.22	0.256	191	0.82	0.71/0.96	1.04	0.87/1.25	0.013
Being a caregiver (n = 106)	No	87	1		1		0.442	139	1		1		0.002
	Yes	17	1.35	0.42/4.29	0.60	0.15/2.36		80	2.56	1.38/4.76	0.85	0.36/2.00	
Chronicity (n = 106)	No chronic disease	16	1		1		0.207	55	1		1		0.013
	With chronic disease	90	0.46	0.12/1.66	1.43	0.29/6.92		164	0.40	0.20/0.79	1.03	0.38/2.75	
Migration status (Country of birth) (n = 106)	Yes (Other country)	13	1		1		0.868	21	1		1		0.968
	No (Spain)	93	1.46	0.36/5.92	1.16	0.28/4.79		201	1.14	0.42/3.10	1.06	0.30/3.64	
Educational level (n = 105)	Primary or less	45	1		1		0.038	115	1		1		0.130
	Secondary or higher education	60	0.48	0.19/1.23	1.70	0.61/4.75		102	1.65	0.91/3.00	0.86	0.41/1.85	
Social risk global assessment (Gijón Questionnaire) (n = 92)	Risk free	52	1		1		0.131	136	1		1		0.023
	At risk or with socio-familial problems	40	0.83	0.30/2.32	2.24	0.80/6.30		55	0.38	0.18/0.79	0.88	0.38/2.02	
Institution (n = 104)	Elderly residence	4	Deleted *				0.146						n.c.
	Prison facilities	42	1.45	0.53/3.95	2.76	0.93/8.17							
	Alcohol withdrawal centre	11	0.60	0.09/4.01	3.45	0.72/16.6							
	No	51	1		1								
Residence area (n = 107)	Rural	78	1		1		0.756	128	1		1		0.896
	Urban or peri-urban	29	0.68	0.24/1.90	0.79	0.28/2.23		89	0.94	0.51/1.71	1.12	0.53/2.36	
Low level of income (n = 102)	Yes	63	1.66	0.64/4.32	4.21	1.42/12.47	0.02	141	0.85	0.45/1.62	2.37	0.88/6.37	0.092
	No	39	1		1			63	1		1		
Live alone (n = 105)	Yes	32	1		1		0.019	52	1		1		0.849
	No	73	4.49	1.50/13.45	1.85	0.68/5.04		167	1.09	0.54/2.20	0.85	0.36/1.99	
Absence of social relationships (n = 104)	Yes	6	0.59	0.09/3.77	0.36	0.03/3.60	0.639						n.c.
	No	98	1		1								
Inadequate housing (n = 106)	Yes	19	1.24	0.39/4.03	0.90	0.24/3.28	0.859	41	0.51	0.23/1.13	1.28	0.54/3.03	0.098
	No	89	1		1			179	1		1		

RRRc: Crude relative risk ratio. n.c. = calculation is not possible due to lack of observations. * In men, those institutionalized in a nursing home were eliminated due to the low number of observations.

**Table 6:** Multivariate multinomial regression models of change in Self-perceived Health for the reference category “Worsening or no improvement” disaggregated.

	**Models with the Whole Group of Men**										**Model with Non-Institutionalized Men in a Nursing Residence**			
	**Model 1**		**Model 2**		**Model 3**		**Model 4**		**Model 5**		**Model 6**			
	**Remained Well**	**Improvement**	**Remained Well**	**Improvement**	**Remained Well**	**Improvement**	**Remained Well**	**Improvement**	**Remained Well**	**Improvement**	**Remained Well**	**Improvement**		
**Variable**	**RRRa** **(CI95% Min/Max)**	**RRRa** **(CI95% Min/Max)**	**RRRa** **(CI95% Min/Max)**	**RRRa** **(CI95% Min/Max)**	**RRRa** **(CI95% Min/Max)**	**RRRa** **(CI95% Min/Max)**	**RRRa** **(CI95% Min/Max)**	**RRRa** **(CI95% Min/Max)**	**RRRa** **(CI95% Min/Max)**	**RRRa** **(CI95% Min/Max)**	**RRRa** **(CI95% Min/Max)**	**RRRa** **(CI95% Min/Max)**		
Age	0.97 * (0.94/0.99)	0.95 * (0.92/0.98)	0.95 * (0.92/0.99)	0.95 * (0.91/0.98)	0.94 * 0.90/0.98)	0.95 * (0.91/0.99)	0.94 * (0.90/0.98)	0.95 * (0.91/0.99)	0.93 * (0.89/0.98)	0.93 * (0.89/0.98)	0.92 * (0.87/0.98)	0.94 * (0.89/1.00)		
Educational level (Secondary or higher education)			0.29 * (0.10/0.83)	0.98 (0.32/3.05)	0.26 * (0.09/0.80)	0.94 (0.29/3.12)	0.25 * (0.08/0.80)	0.89 (0.27/2.99)	0.21 * (0.07/0.69)	0.80 (0.23/2.77)	0.2 * (0.06/0.68)	0.89 (0.25/3.19)		
Low income (yes)					0.72 (0.23/2.27)	2.09 (0.61/7.15)	0.71 (0.21/2.38)	1.98 (0.57/6.92)			1.34 (0.34/5.28)	3.4 ** (0.81/14.21)		
Live alone (no)							5.11 * (1.52/17.1)	2.02 (0.67/6.14)			5.2 * (1.49/18.03)	2.06 (0.65/6.59)		
Social risk global assessment (Gijón Questionnaire) (at risk)									0.35 (0.10/1.22)	0.90 (0.26/3.11)				
Institutionalized in a prison facility (yes)											0.24 (0.04/1.53)	0.42 (0.07/2.45)		
Institutionalized in an alcohol withdrawal centre (yes)											0.17 (0.02/1.75)	0.68 (0.09/5.19)		
	**Models in Women**													
	**Model 1**		**Model 2**		**Model 3**		**Model 4**		**Model 5**		**Model 6**		**Model 7**	
	**Remained Well**	**Improvement**	**Remained Well**	**Improvement**	**Remained Well**	**Improvement**	**Remained Well**	**Improvement**	**Remained Well**	**Improvement**	**Remained Well**	**Improvement**	**Remained Well**	**Improvement**
**Variable**	**RRRa** **(CI95% min/max)**	**RRRa** **(CI95% min/max)**	**RRRa** **(CI95% min/max)**	**RRRa** **(CI95% min/max)**	**RRRa** **(CI95% min/max)**	**RRRa** **(CI95% min/max)**	**RRRa** **(CI95% min/max)**	**RRRa** **(CI95% min/max)**	**RRRa** **(CI95% min/max)**	**RRRa** **(CI95% min/max)**	**RRRa** **(CI95% min/max)**	**RRRa** **(CI95% min/max)**	**RRRa** **(CI95% min/max)**	**RRRa** **(CI95% min/max)**
Age	0.98 (0.96/1.01)	0.98 (0.95/1.00)	1.00 (0.98/1.02)	0.97 ** (0.94/1.00)	1.00 (0.98/1.03)	0.97 ** (0.94/1.00)	1.00 (0.97/1.03)	0.96 * (0.96/1.00)	1.00 (0.97/1.03)	0.95 * (0.92/0.99)	1.00 (0.98/1.03)	0.96 * (0.93/1.00)	1.00 (0.97/1.03)	0.96 * (0.93/1.00)
Is a caregiver (yes)			2.59 * (1.29/5.20)	0.55 (0.21/1.47)	2.09 (0.79/5.48)	0.52 (0.12/2.16)	1.88 (0.68/5.25)	0.29 (0.06/1.58)	1.85 (0.66/5.21)	0.25 (0.05/1.39)	1.82 (0.68/4.95)	0.45 (0.10/1.93)	1.84 (0.68/5.03)	0.44 (0.10/1.94)
Chronicity (yes)					0.71 (0.25/2.04)	0.89 (0.18/4.44)	0.71 (0.23/2.15)	0.62 (0.10/3.93)	0.76 (0.25/2.37)	0.53 (0.08/3.50)	0.71 (0.24/2.14)	0.70 (0.14/3.63)	0.72 (0.24/2.17)	0.92 (0.17/4.94)
Social risk global assessment (Gijón Questionnaire) (at risk)							0.38 * (0.17/0.80)	0.63 (0.10/3.93)	0.36 * (0.17/0.78)	0.67 (0.27/1.64)				
Educational level (Secondary or higher education)									1.19 (0.56/2.54)	0.62 (0.23/1.68)	1.31 (0.64/2.68)	0.60 (0.28/1.50)	1.22 (0.58/2.59)	0.83 (0.32/2.19)
Inadequate housing (yes)											0.50 (0.22/1.14)	0.94 (0.37/2.36)	0.53 (0.23/1.24)	0.93 (0.35/2.46)
Low income (yes)													0.82 (0.41/1.63)	1.89 (0.68/5.30)

RRRa: Adjusted relative risk ratio. Reference categories male models: primary educational level or less; no low income; yes, live alone; not social risk; not institutionalized. Reference categories female models: non-caregiver, non-chronic, non-social risk; primary educational level or less; no inadequate housing; not low income. * *p* value < 0.05 ** *p* value 0.05–0.1.

## Data Availability

The study protocol was registered through the ISRCTN Registry (Ref. ISRCTN70517103) on 20 June 2018. Data can be requested through the contact information provided there: Pisano González, M. and Boone, A.L. (2018) ‘EFFICHRONIC: Efficiency analysis of a self-management programme in 5 European countries, in vulnerable people with chronic diseases and/or their caregivers’. Available at https://doi.org/10.1186/ISRCTN70517103.

## Supplementary Materials

The following supporting information can be downloaded at: https://www.mdpi.com/article/10.3390/healthcare12080811/s1, Supplementary Table S1: Recategorization of items from the Gijón Scale; Supplementary Table S2. Global bivariate for change in SPH; Supplementary Table S3. Bivariate analysis for change in SPH in men; Supplementary Table S4. Bivariate for change in SPH in women; Figure S1. Summary multinomial regression change in SPH: Global data; Figure S2. Summary multinomial regression change in SPH: Men; Figure S3. Summary multinomial regression change in SPH: Women.
