# Supplementary material for: Impact of Chronic Disease Self-Management Program on the Self-Perceived Health of People in Areas of Social Vulnerability in Asturias, Spain
Source: Healthcare (Basel). 2024 Apr 9;12(8):811. doi: 10.3390/healthcare12080811 (PMC11049834; doi:10.3390/healthcare12080811)
Supplement: Supplementary file 1 [file healthcare-12-00811-s001.zip › healthcare-2905561-supplementary.pdf]

Supplementary Table S1: Recategorization of items from the Gijón Scale

Scale item	Recategorization	Included categories
Low level of income<br>(choose only one answer, the most suitable)	Yes	From 707 to 1060 euros per month.<br>From 443 to 706 euros per month.<br>I receive a non-contributory pension, for disability or social assistance.<br>Less than the previous sections or I have no income)
	No	More than 1060 euros per month
Living alone	Yes	I live alone and I am independent.<br>I live alone with close family and have some degree of dependency.<br>I live alone and isolated and have some degree of dependency.
	No	I live accompanied with or without dependence
Absence of social relationships<br>(choose only one answer, the most suitable)	Yes	I don't leave the house or receive visitors (or less than once a week)
	No	I maintain social relationships outside the home with many people.<br>I interact with family and neighbors/friends and leave the house.<br>I only interact with family and leave the house.<br>I don't leave the house, but I receive visitors (at least once a week)
Absence of social support<br>What type of social support do you have?<br>(choose only one answer, the most suitable)	Yes	I need permanent care that is not given.
	No	I don't need any support.<br>I receive support from family and/or neighbors/friends.<br>I receive formal or voluntary home help.<br>I receive help in a residence.
Inadequate housing<br>(choose only one answer, the most suitable)	Yes	Has architectural barriers inside or at the entrance of the house.<br>It has humidity (or similar problems) or is not adequately equipped (incomplete bathroom, absence of hot water, heating, ...)<br>Absence of elevator (if living in an apartment) or telephone.<br>It is inadequate: a shanty, declared in ruins, lack of minimal equipment, overcrowded, …
	No	It is suitable for my needs.

Source: (Cabrera González et al., 1999)

Supplementary Table S2. Global bivariate for change in SPH

		GLOBAL		WORSENING n=62 (18.7%)		NO IMPROVEMENT n=65 (19.6%)		REMAINED WELL n=129 (38.9%)		IMPROVEMENT n=76 (22.9%)		P-VALUE
**VARIABLES**		Mean (SD)		Mean (SD)		Mean (SD)		Mean (SD)		Mean (SD)		
**AGE (n=330)**		60.5 (15.1)		62.2 (14.7)		66 (13.5)		59.7 (15.1)		55.8 (15.2)		0.756
**SOCIAL RISK (Continuous variable*) (n=283)**		8.70 (2.6)		8.0(2.6)		9.9 (2.8)		8.1 (2.2)		9.4 (2.5)		0.140
**Variable**	**Categories (base code in front)**	n	%	n	%	n	%	n	%	n	%	
**Participant type (n=326)**	**Chronic**	227	69.6	40	17.6	52	22.9	75	33.0	60	26.4	0.001
	**Caregiver**	71	21.8	15	21.1	5	7.0	41	57.8	10	14.1	
	**Both**	28	8.59	3	10.7	8	28.6	13	46.4	4	14.3	
**Sex (n=331)**	**Man**	108	32.6	19	17.6	16	14.8	39	36.1	3. 4	31.5	0.065
	**Women**	223	67.4	43	19.3	48	21.5	90	40.4	42	18.8	
**MIGRATION STATUS (Country of birth) (n=329)**	**Yes (Other country)**	3. 4	10.3	9	26.5	5	14.7	12	39.7	8	23.5	0.62
	**No (Spain)**	295	89.7	53	18.0	59	20	117	35.3	66	22.4	
**EDUCATIONAL LEVEL** (three categories) (n=323)	**Primary or less**	161	49.9	23	14.3	42	26.1	62	38.5	3. 4	21.1	0.008
	**Secondary**	121	37.5	23	19.0	16	13.2	47	38.8	35	28.9	
	**University students**	41	12.7	13	31.7	6	14.6	18	43.9	4	9.8	
**RESIDENCE INSTITUTION (n=332)**	**None**	261	78.6	49	18.8	53	20.3	110	42.2	49	18.8	0.004
	**Nursing home**	7	2.1	3	42.9	3	42.9	0	0	1	14.3	
	**Prison facility**	47	1.95	8	17.0	6	12.8	16	34.0	17	36.2	
	**Alcohol withdrawal center**	17	5.1	2	11.8	3	17.7	3	17.7	9	52.9	
**RESIDENCE AREA (n=325)**	**Rural Peasant**	75	23.1	17	22.7	11	14.7	30	40	17	22.7	0.148
	**Rural Intensive**	131	40.3	16	12.2	33	25.2	51	38.9	31	23.7	
	**Peri urban Peasant**	41	12.6	13	31.7	10	24.4	12	29.3	6	14.6	
	**Intensive Peri urban**	42	12.9	7	16.7	5	11.9	20	47.6	10	23.8	
	**Urban**	36	2.6	8	22.2	6	16.7	11	30.6	11	30.6	
**SOCIAL RISK GLOBAL ASSESSMENT (Gijón Questionnaire)** *(n=283)*	**Risk free**	188	66.4	42	22.3	24	12.8	87	46.3	35	18.6	0.000
	**With socio-familial risk**	88	31.1	9	10.2	26	29.6	26	29.6	27	30.7	
	**Socio-familial problems**	7	2.5	2	28.6	3	42.9	0	0	2	28.6	
**SOCIAL RISK GLOBAL ASSESSMENT (Gijón Questionnaire)** *(n=283)*	**Risk free**	188	66.4	42	22.3	24	12.8	87	46.3	35	18.6	0.000
	**At risk/with socio-familial problems**	95	33.6	11	11.6	29	30.5	26	27.4	29	30.5	
**VARIABLES OF THE GIJÓN QUESTIONNAIRE DICHOTOMIZED**		n	%	n	%	n	%	n	%	n	%	
**LOW LEVEL OF INCOME** *(n=307)*	**Yes**	205	66.8	27	13.2	46	22.4	76	37.1	56	27.3	0.001
	**No**	102	33.2	28	27.5	16	15.7	45	44.1	13	12.8	
**LIVE ALONE (n=324)**	**Yes**	84	25.9	16	19.1	21	25.0	26	31.0	21	25	0.217
	**No**	240	74.1	45	18.8	41	17.1	102	42.5	52	21.7	
**ABSENCE OF SOCIAL RELATIONSHIPS** *(n=326)*	**Yes**	6	1.8	1	16.7	2	33.3	2	33.3	1	16.7	0.854
	**No**	320	98.2	61	19.1	61	19.1	126	39.4	72	22.5	
**ABSENCE OF SOCIAL SUPPORT** *(n=312)*	**Yes**	2	0.6	0	0.0	1	50.0	0	0.0	1	50.0	0.419
	**No**	310	99.4	60	19.4	58	18.7	121	39.0	71	22.9	
**INADEQUATE HOUSING** *(n=327)*	**Yes**	61	18.7	7	11.5	19	31.2	19	31.2	16	26.2	0.025
	**No**	266	81.4	54	20.3	44	16.5	110	41.4	58	21.8	

* Gijón Scale: minimum score =5 and maximum =25 points. Sections: <10 indicates no socio-familial risk, 10 to 14 in risk, >15 problems

Supplementary Table S3. Bivariate analysis for change in SPH in men

		GLOBAL		WORSENING (n=19; 17.6%)		NO IMPROVEMENT (n=16; 14.8%)		REMAINED WELL (n=39; 36.1%)		IMPROVEMENT (n= 34; 31.5%)		P-VALUE
**VARIABLES**		**Mean (SD)**		**Mean (SD)**		**Mean (SD)**		**Mean (SD)**		**Mean (SD)**		
**AGE (n=107)**		55.7 (16.8)		63.3(16.0)		62.1(16.5)		54.3(17.9)		50.0(13.7)		0.504
**SOCIAL RISK (Continuous *) (n=92)**		9.2 (3.2)		8.4 (3.7)		10.5(4.0)		8.5 (2.7)		9.8 (2.8)		0.227
**Variable**	**Categories (base code in front)**	**n**	**%**	**n**	**%**	**n**	**%**	**n**	**%**	**n**	**%**	
**Participant type (n=106)**	**Chronic**	16	15.1	14	16.9	13	14.9	30	34.5	30	34.5	0.102
	**Caregiver**	87	82.1	3	18.8	1	6.25	9	56.3	3	18.8	
	**Both**	3	2.8	0	0.0	2	66.7	0	0.0	1	33.3	
**MIGRATION STATUS (Country of birth) (n=106)**	**Yes (Other country)**	13	12.3	5	38.5	0	0.0	4	30.8	4	30.8	0.118
	**No (Spain)**	93	87.7	14	15.1	16	17.2	35	37.6	28	30.1	
**EDUCATIONAL LEVEL** (n=105)	**Primary or less**	45	42.9	6	13.3	8	17.8	22	48.9	9	20	0.041
	**Secondary**	47	44.8	8	17.0	5	10.6	14	29.8	20	42.6	
	**University students**	13	12.4	5	38.5	3	23.1	2	15.4	3	23.1	
**RESIDENCE INSTITUTION (n=108)**	**None**	51	47.2	11	21.6	8	15.7	21	41.2	11	21.6	0.189
	**Nursing home**	4	3.7	1	25.0	2	50	0	0	1	25.0	
	**Prison facility**	42	38.9	6	14.3	4	9.5	16	38.1	16	38.1	
	**Alcohol withdrawal center**	11	7.5	1	9.1	2	18.2	2	18.2	6	54.6	
**RESIDENCE AREA (n=107)**	**Rural Peasant**	21	19.6	5	23.8	2	9.5	7	33.3	7	33.3	0.600
	**Rural Intensive**	87	81.3	9	15.8	8	14.0	22	38.6	18	32.6	
	**Peri urban Peasant**	8	7.5	1	12.5	3	37.5	3	37.5	1	12.5	
	**Intensive Peri urban**	8	7.5	2	25	1	12.5	4	50.0	1	12.5	
	**Urban**	13	12.1	2	15.4	2	15.4	2	15.4	7	54.9	
**SOCIAL RISK GLOBAL ASSESSMENT (Gijón Questionnaire)** (n=283)	**Risk free**	52	56.5	12	23.1	7	13.5	21	40.4	12	23.1	0.086
	**At risk/with socio-familial problems**	40	43.5	4	10	8	20	11	27.5	17	42.5	
**VARIABLES OF THE GIJÓN QUESTIONNAIRE DICHOTOMIZED**		**n**	**%**	**n**	**%**	**n**	**%**	**n**	**%**	**n**	**%**	
**LOW LEVEL OF INCOME** (n=102)	**Yes**	63	61.8	6	9.5	9	14.3	22	34.9	26	41.3	0.040
	**No**	39	38.2	10	25.6	7	18.0	15	38.5	7	18.0	
**LIVE ALONE (n=105)**	**Yes**	32	30.5	6	18.8	10	31.3	6	18.8	10	31.3	0.009
	**No**	73	69.5	13	17.8	6	8.2	32	43.8	22	30.1	
**ABSENCE OF SOCIAL RELATIONSHIPS** (n=104)	**Yes**	6	5.8	1	16.7	2	33.3	2	33.3	1	16.7	0.630
	**No**	98	94.2	18	18.4	14	14.3	36	36.7	30	30.6	
**ABSENCE OF SOCIAL SUPPORT** (n=99)	**Yes**	2	2.0	0	0.0	1	50.0	0	0.0	1	50.0	0.398
	**No**	97	98.0	19	19.6	14	14.4	3. 4	35.1	30	30.9	
**INADEQUATE HOUSING** (n=106)	**Yes**	19	17.9	3	15.8	3	15.8	8	42.1	5	26.3	0.949
	**No**	87	82.1	16	18.4	13	14.9	31	35.6	27	31.0	

* Gijón Scale: minimum score =5 and maximum =25 points. Sections: <10 indicates no socio-familial risk, 10 to 14 in risk, >15 problems

Supplementary Table S4. Bivariate for change in SPH in women

		GLOBAL		WORSENING (n= 43; 19.3%)		NO IMPROVEMENT (n= 48; 21.5%)		REMAINED WELL (n= 90; 40.4%)		IMPROVEMENT (n= 42; 18.8%)		P-VALUE
**VARIABLES**		**Mean (SD)**		**Mean (SD)**		**Mean (SD)**		**Mean (SD)**		**Mean (SD)**		
**AGE (n=222)**		62.8 (13.7)		61.7 (14.2)		67.5 (12.3)		62.0 (13.1)		60.4 (14.8)		0.598
**SOCIAL RISK (Continuous*) (n=191)**		8.4 (2.2)		171 (2.0)		9.7 (2.2)		7.9 (1.9)		8.9 (2.2)		0.677
**Variable**	**Categories**	**n**	**%**	**n**	**%**	**n**	**%**	**n**	**%**	**n**	**%**	
**Participant type (n=219)**	**Chronic**	139	63.5	26	18.7	38	27.3	45	32.4	30	21.6	0.007
	**Caregiver**	55	25.1	12	21.8	4	7.3	32	58.2	7	12.7	
	**Both**	25	11.4	3	12.0	6	24.0	13	52.0	3	12.0	
**MIGRATION STATUS (Country of birth) (n=222)**	**Yes (Other country)**	21	9.5	4	19.1	5	23.8	8	38.1	4	19.1	0.999
	**No (Spain)**	201	90.5	39	19.4	42	20.9	82	40.8	28	18.9	
**EDUCATIONAL LEVEL** (n=217)	**Primary or less**	115	53	17	14.8	33	28.7	40	34.8	25	21.7	0.017
	**Secondary**	74	34.1	15	20.3	11	14.9	33	44.6	15	20.3	
	**University students**	28	12.9	8	28.6	3	10.7	16	57.1	1	3.6	
**RESIDENCE INSTITUTION (n=223)**	**None**	209	93.7	38	18.2	44	21.1	89	42.6	38	18.2	0.127
	**Nursing home**	3	1.3	2	66.7	1	33.3	0	0	0	0	
	**Prison facility**	5	2.2	2	40	2	40	0	0	1	20	
	**Alcohol withdrawal center**	6	2.7	1	16.7	1	16.7	1	16.7	3	50	
**RESIDENCE AREA (n=217)**	**Rural Peasant**	54	24.8	12	22.2	9	16.7	23	**42.6**	10	18.5	0.056
	**Rural Intensive**	74	3.1	7	9.5	25	33.8	29	**39.2**	13	17.6	
	**Peri urban Peasant**	32	14.7	12	37.5	6	18.8	9	**28.1**	5	15.6	
	**Intensive Peri urban**	3. 4	15.7	5	14.7	4	11.8	16	**47.1**	9	26.5	
	**Urban**	23	10.6	6	26.1	4	17.4	9	**39.1**	4	17.4	
**SOCIAL RISK GLOBAL ASSESSMENT (Gijón Questionnaire*) (n=283)**	**Risk free**	136	71.2	30	22.1	17	12.5	66	48.5	23	16.9	0.000
	**At risk/with socio-familial problems**	55	28.8	7	12.7	21	38.2	15	27.3	12	21.8	
**VARIABLES OF THE GIJÓN QUESTIONNAIRE DICHOTOMIZED**		**n**	**%**	**n**	**%**	**n**	**%**	**n**	**%**	**n**	**%**	
**LOW LEVEL OF INCOME** (n=204)	**Yes**	141	69.1	21	14.9	36	25.5	54	38.3	30	21.3	0.011
	**No**	63	30.9	18	28.6	9	14.3	30	47.6	6	9.5	
**LIVE ALONE (n=219)**	**Yes**	52	23.7	10	19.2	11	21.2	20	38.5	11	21.2	0.954
	**No**	167	76.3	32	19.2	35	21.0	70	41.9	30	18.0	
**ABSENCE OF SOCIAL RELATIONSHIPS** (n=221)	**Yes**	0	0.0	0	0.0	0	0.0	0	0.0	0	0.0	n. c.
	**No**	221	100	43	19.5	46	20.8	90	40.7	42	19	
**ABSENCE OF SOCIAL SUPPORT** (n=212)	**Yes**	0	0.0	0	0.0	0	0.0	0	0.0	0	0.0	n. c.
	**No**	212	100	41	19.3	43	20.3	87	41.0	41	19.3	
**INADEQUATE HOUSING** (n=220)	**Yes**	41	18.6	4	9.8	15	36.6	11	26.8	11	26.8	0.007
	**No**	179	81.4	38	21.2	31	17.3	79	44.1	31	17.3	

* Gijón Scale: minimum score =5 and maximum =25 points. Sections: <10 indicates no socio-familial risk, 10 to 14 in risk, >15 problems.; n. c. = calculation is not possible due to lack of observations.

Figure S1. Summary multinomial regression change in self-perceived health: Global data.

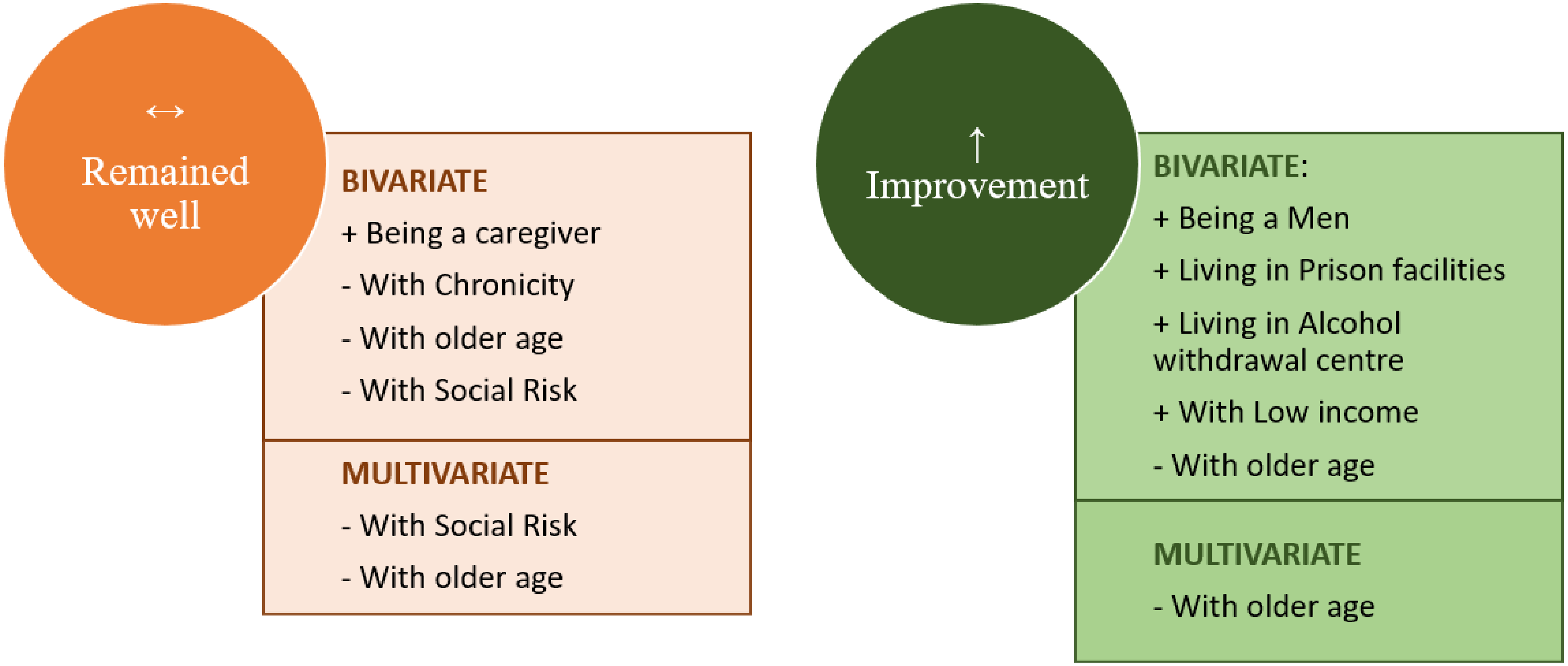

+: increases the probability versus "worsening or no improvement"

-: decreases the probability versus "worsening or no improvement"

Figure S2. Summary multinomial regression change in self-perceived health: Men.

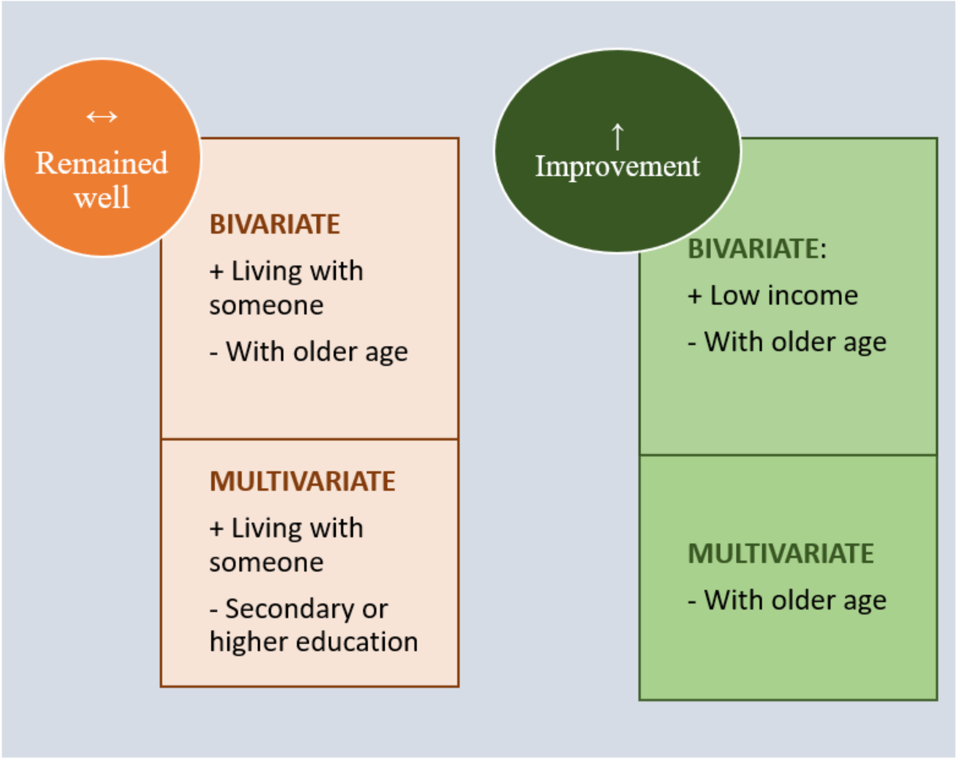

+: increases the probability versus "worsening or no improvement"
-: decreases the probability versus "worsening or no improvement"

Figure S3. Summary multinomial regression change in self-perceived health: Women.

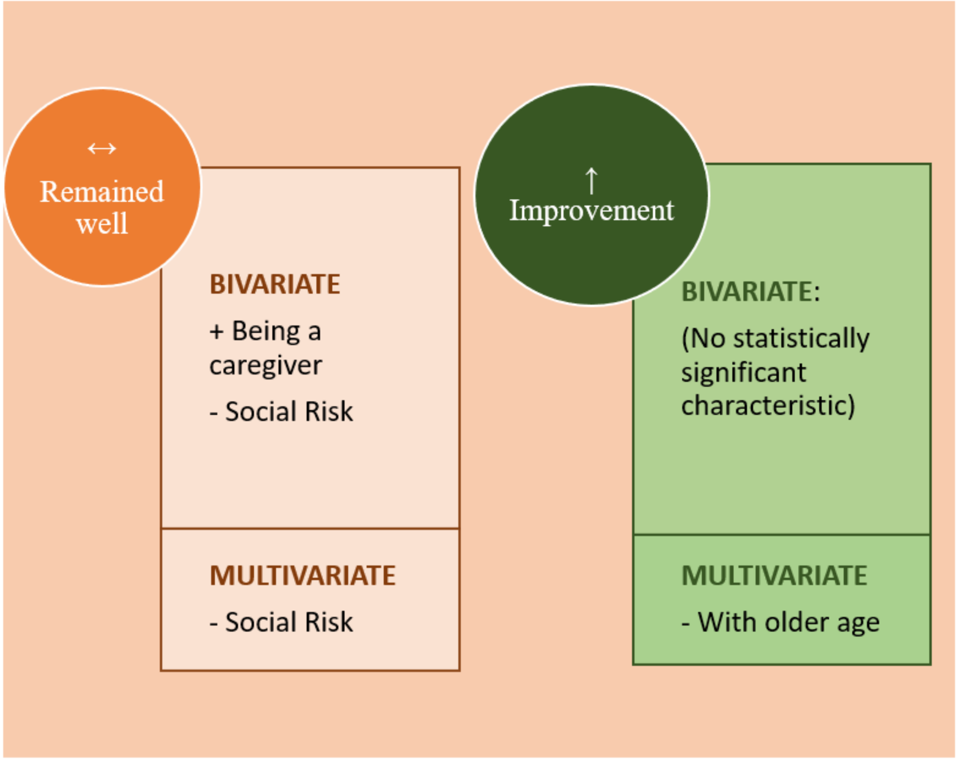

+: increases the probability versus "worsening or no improvement"
-: decreases the probability versus "worsening or no improvement"
